# Pennsylvania Medical College, Philadelphia

**Published:** 1843-10

**Authors:** 


### PENNSYLVANIA MEDICAL COLLEGE, PHILADELPHIA.

Difficulties, it is said, have occurred among the Faculty of this College, in consequence of which they have resigned. It is not known whether the school will be re-organized or not; but at such a period of the year it is improbable that any arrangement can be made to insure a course of lectures the coming winter. The Faculty embraced some distinguished men.

What the poet says of human affairs generally, is particularly applicable to things medical and at the same time collegiate:

"Naught may endure but Mutability."

